# Social relations and exclusion among people facing death

**DOI:** 10.1007/s10433-023-00749-y

**Published:** 2023-01-31

**Authors:** Marjaana Seppänen, Mia Niemi, Sofia Sarivaara

**Affiliations:** University of Helsinki, Helsinki, Finland

**Keywords:** Death, Dying, Social relationships, Family, Exclusion

## Abstract

In line with current policies and service developments related to palliative care, more people are dying at home. This situation has provoked discussions about the importance of non-medical issues related to death. The process of dying is often long, with many phases, and the social aspect is a major part of it. Our focus in this article is on dying as a social process. Social relationships are significant and play a meaningful role in enhancing the well-being of older adults approaching the end of life. Meaningful social relationships tend to change over time; however, and the process of dying may exacerbate such changes in and challenge these relationships. The aim of our study was to examine how social relationships are experienced and (re)constructed among older adults (70–83 years old) during the process of dying, in a Finnish context. We were interested in the nature and type of these relationships, and in the possible new forms of expression that may emerge during this process. Our empirical data were based on interviews with seven older adults who were close to death. The analysis revealed processes of exclusion from existing relations. At the same time, we observed new and unexpected relations being initiated, which sometimes became meaningful and supportive. The results highlighted the role of expectations and importance of analysing exclusion from a life-course perspective.

## Introduction

### Death returns home

The number of patients receiving home-based end-of-life care has been increasing in European countries, including Finland, in recent decades. Among Western societies, the place of death is influenced by individual factors such as age, cause of death and ethnic origin, and social factors such as the organisation of services and cultural expectations (Lloyd 2010). Keeping people at home in their old age in spite of illness is an explicit policy goal in Finland (Outila et al. 2019). Even though these ageing- or dying-in-place policies include multiple contradictory aspects (Sixsmith and Sixsmith 2008), in the view of many people the home is, in fact, the preferred option. Nevertheless, the most common place of death in Finland is in a health facility (Forma et al. 2020).

Death became medicalised and institutionalised during the transition from a traditional to a modern society (Exley 2004). The place of dying shifted from homes to hospitals and institutions, and death became isolated from everyday life (Miettinen 2006; Elias 1985). In line with current policies and service developments related to palliative care, death has “returned” home (Outila et al. 2019; Jeppsson Grassman and Whitaker 2007). This situation has provoked discussions on the importance of non-medical issues related to death, and questions regarding what constitutes a good death have been posed anew. The need for a holistic view of the dying, which emphasises the psychological, social and spiritual needs of individuals, has become acute (Lloyd 2010). Moreover, scholars have suggested broader conceptualisations of the end of life (Bern-Klug 2004), and have proposed social models for end-of-life care (Brown and Walter 2014).

An understanding of dying as a social process, and of the home as the context of that process, highlights the importance of informal care. Informal care is often provided by family members, for example partners or adult children (Åkerman et al 2021). However, the social aspect of dying includes more than the care dimension, which nevertheless tends to predominate. Social relationships are meaningful for older adults, and constitute a substantial part of well-being and fulfilment in life (Burholt and Aartsen 2021), even to those approaching its end. Hence, the social aspect of dying is an important part of the process, which may be long with many phases. Moreover, the process of dying may cause changes in and challenge meaningful social relationships, which changes over time.

### Dying as a social process

Time and approaching the end of life are significant factors in the ageing process, as is acknowledged in the theoretical discussion within the field of social gerontology (Lloyd 2010). Despite their proximity in old age, research on death and dying has not attracted as much attention as could be expected. However, death-related research has gradually expanded in recent decades and there is growing scholarly interest in the phenomenon of dying, which is increasingly likely to occur at a very advanced age. Contemporary research has investigated palliative care, with a focus on death in nursing homes and other institutions (Macgregor et al. 2021; Ullrich et al. 2019), and an emphasis on the roles and perspectives of professionals. The Covid-19 pandemic further induced research into end-of-life-care (Mitchell et al. 2021; Porter et al. 2021). The role of informal relationships has also been discussed in this context, although mainly in relation to formal care. Nevertheless, the literature on the sociology of death has been expanding, with contributions initially coming from multi-disciplinary teams (Howarth 2007; Kellehear 2008; Holmberg et al. 2019).

Although the idea of a good death is understood to comprise a holistic view of the dying, including their psychological, social and spiritual needs (Lloyd 2010), scholars point out that the “disadvantaged dying” (such as older people without access to sufficient support) need more attention in research (Exley 2004). It has been found in some studies that social support is directly connected to the fear of death: when it is available, there is less anxiety and fear regarding death and dying (Chopik 2017; Cicirelli 1999, 2002). Lloyd et al. (2011) conclude that relationships with family and friends are essential to a good or “bad” death. The availability of social support for the dying may relate to lifelong circumstances, but there may also be changes in social relationships just before death. This paper focuses on changes in social relationships over the course of dying, from the conceptual perspective of social exclusion. This paper focuses on social relationships in the context of dying, and especially on changes in them, from the conceptual perspective of exclusion.

### Social exclusion and exclusion from social relationships

In recent years, the concept of social exclusion has been developed in connection with research on older adults. Exclusion has been conceptualised in different yet related ways, but the key elements in the definitions are its relative and dynamic nature. Consequently, exclusion should be seen in the context of the society in which people live and as something that changes over time (e.g. Walsh et al. 2021; Macleod et al. 2019). The definition suggested by Levitas et al. (2007: 25; see also Walsh et al. 2021, 11) reflects these key elements: a lack or the denial of resources, rights, goods and services, and the inability to participate in the normal relationships and activities that are available to the majority of people in a society.

The concept of exclusion has been criticised on the grounds of a lack of clarity in connection with operationalisation, but it is argued that its strength lies in the focus on processes rather than outcomes, which in turn enhances understanding of inequalities and social injustice (Sealey 2015). At the core of the concept is multidimensionality: exclusion may operate in different (interrelated) domains, one or several at the same time. The key domains of social exclusion in different definitions (see Walsh et al. 2021; Keating and Scharf 2012) include economic circumstances, social relations, services, community & spatial issues and civic exclusion. The focus in empirical research tends to be on one of them, but taking into consideration the multidimensional nature of the phenomenon. Although in the context of political discourse exclusion is often connected to economic difficulties, exclusion from social relations has been strongly present in conceptual discussion (Waldegrave et al. 2021; Walsh et al. 2021; MacLeod et al. 2019; Burholt et al. 2017; Keating and Scharf 2012; Walsh et al. 2016).

Being excluded from social relations touches on the fundamental aspects of social life and is considered a serious threat to well-being (Burholt and Aartsen 2021; Waldegrave et al 2021; Vaara et al. 2016). Such exclusion has been defined as follows:

… we define exclusion from social relations as a situation in which people are disconnected from adequate levels of quality of intimate relationships, social networks, social support, and/or social opportunities to participate in wider society. (Burholt and Aartsen 2021, 77)

Even though key events that are more likely to occur in old age, such as the death of family members or friends, increase the risk of exclusion (MacLeod et al. 2019), a life-course approach is important (Van Regenmortel 2017) in analyses of exclusion from social relations. However, its potential remains underutilised (Burholt and Aartsen 2021). It would be useful, for instance, to see and understand people’s current social needs and relationships in relation to the life course, and to recognise the impact of potential adversities experienced throughout the lived life (Ejlskov et al. 2019; Tiilikainen and Seppänen 2017).

Potential drivers have been identified in the theoretical development of the concept of exclusion in later life. According to Scharf and Keating (2012, 6–9), key drivers operate on three different levels. First, structural drivers (such as ageism, discrimination and both social and economic policies) can be identified on the national level; second, environmental drivers relate to the living environment of older persons and include aspects such as the changing nature of urban communities; third, examples of individual-level drivers include a migratory life course, disruption in social networks and ill-health (see also De Jong Gierveld et al. 2009; Aartsen et al. 2004).

As stated above, the main aspects of exclusion as a concept are multidimensionality and relativity. Another key element is its dynamic nature. In the context of exclusion from social relationships, for example, we believe that the aspect of process is paramount. A more contested although less prominent notion is the involvement of agency, in other words that individuals, consciously or sub-consciously, may choose to exclude themselves (Walsh et al. 2021). The concept of *solitude*, or voluntary withdrawal, was developed earlier to describe situations in which individuals voluntarily decide to withdraw from social connections (Nguyen et al. 2017). In that exclusion tends to be understood as a negative phenomenon, it differs from the concept of solitude, and rather connotates the idea of *social death* with its implications of a loss of social identity and connectedness (Králová 2015; see also Pirhonen et al. 2021). Just as losing all social contacts could be referred to as social death while living, living “in social death” could also result in dying alone without conscious intention, or even choosing to die alone (Caswell and O´Connor 2015). Our focus in this article is on exclusion understood as an unvoluntary state of being.

A recent literature review (ten Bruggencate et al. 2018) focusing on the social needs of older people divided the analysed empirical articles according to the themes of diversity, proximity, the meaning of relationships and reciprocity. The relationships of older adults were described as (i) intimate relationships that bring love and belonging and (ii) peripheral relationships (such as contacts at clubs, churches and pubs) that make older adults feel part of life and of society.

Both types (intimate and peripheral) of social relations could be considered essential to well-being. However, it is argued that family relationships are at the core of the domain of exclusion from social relationships (Keating and Scharf 2012). Discussions in Europe have emphasized—in addition to the centrality of family relationships—the quality and complexity of family interactions. The idea of family has undergone a transformation and new kinds of familial patterns prevail (Jallinoja and Widmer 2011). Families are constellations of individuals that change continuously and could be understood as the result of negotiated family practices (Chambers et al. 2009), which highlights the importance of relations and relatedness in research on families. Older adults define social relations, especially family relations, and health as the key factors of well-being (Vaara et al. 2016). Consequently, exclusion from social relations could be a major threat.

This article is based on the understanding that exclusion from social relationships as a phenomenon is connected to reduced social opportunities, a low quality of social relationships, a lack of social networks and support, and feelings of isolation and loneliness (Walsh et al. 2016). Both the quantity and quality of social relations matter (Burholt and Aartsen 2021; Burholt et al. 2017). Whether or not approaching death influences the exclusion of the dying person is an interesting and underexplored question. In our study, therefore, we examined the kinds of social relations a dying older adult has, and how approaching death changes such relationships. We did not focus on care arrangements or palliative care as such, even though they are strongly present in the lives of dying older adults.

## The research question

The aim of our study was to examine how the social relationships of older adults are constituted and how they change during the process of dying. We were interested in the nature and types of relationship, and in the possible new ways in which social relations are expressed during this process. We focused specifically on changes that occur when death is approaching, which we analysed from the exclusion perspective to shed light on how dying affects exclusion from existing relations.

The research questions were as follows:

1. What kind of social relationships do dying people have?
2. What changes in social relationships occur during the process of dying?

We were interested specifically in the relationships that dying older adults have with family members, relatives, friends, acquaintances, neighbours and people related to work and hobbies. In other words, we examined both intimate and peripheral relationships (see ten Bruggencate et al. 2018).

## Data and methods

Our empirical data, collected in Finland, consisted of semi-structured interviews with seven older adults (aged 70–83 years) who were approaching death. The data were collected as part of a multidisciplinary study on the meaningful relationships of older adults (MeRela) in palliative care following a decision made by a doctor, or who had been diagnosed with an illness that would lead to death and who were spending their final days in a private home. The participants were recruited via private hospices and a public at-home hospital network in southern Finland (see also Saarelainen et al. 2020). Social and health care workers, typically nurses, inquired whether their clients would be interested in participating in the study. With the clients’ permission, the workers then passed on the relevant contact information to the research group. A researcher contacted the potential participant to give more information about the study and to discuss the interview in more detail. In addition, an announcement about the study was published in an online and print newspaper of the Evangelical Lutheran Church of Finland in the Helsinki area, which made it possible for potential participants to reach out if they wished to contribute. A few participants were also contacted using the snowball method.

The semi-structured interviews were carefully planned to integrate the different backgrounds of the multidisciplinary research team that included representatives of theology, nursing, social sciences and law. Two interviewers conducted the interviews, which allowed researchers with different disciplinary backgrounds to participate. One of them assumed the role of primary interviewer in acknowledgement of the power dynamics of an interview. The interviews were conducted in pairs because they were potentially emotionally draining for the interviewers, given that the interviewees were facing death (Valentine 2007). The two researchers had a reflexive discussion after each interview to ensure that ethical aspects were considered throughout the process, during which they also evaluated the participants’ need for support.

Each interview covered the following main themes: life here and now following the palliative-care decision, the home as an environment, social relationships, life before the illness, services and support, values and worldview, personal rights and the narrative of the future. In addition to the thematic interview, we used visual aids (Pictor: see, e.g. King et al. 2013) as a tool (Saarelainen et al. 2020). However, the drawings were not part of the data analysed in this article: we used the Pictor method only as part of the interviews to define how the participants located their social relationships.

This article is based on seven interviews with dying older adults, two males and five females. The data are part of a total of 32 interviews that were conducted for the research project. The participants of this study were in palliative care, or had been diagnosed/living with a terminal illness but still coped primarily independently at home. Three of them lived with their spouse and four lived alone. All except one had adult children with whom they frequently interacted.

The participants were assigned pseudonyms to ensure confidentiality and anonymity, and any details that could jeopardise their anonymity were left out. The interviews were conducted in Finnish and the quotations were translated into English for the purposes of this article: every attempt was made to preserve the original form of expression.

For the purposes of this article we used the phenomenological-hermeneutical method to facilitate understanding and interpretation of the participants’ lived experiences (Laverty 2003). Our aim was to achieve an in-depth understanding of the meaning that dying older adults attribute to social relationships, and the changes that such relationships undergo during the dying process.

Using Atlas TI, we subjected the transcribed interviews to thematic analysis (Nowell et al. 2017). First, we identified the participants’ social relationships and coded them accordingly. Next, we grouped the social relationships into different categories to describe the nature of the relationships. Then we analysed the data descriptions and the expressions connected to the meanings and changes in the relationships. We identified essential topics and themes, dividing them according to our analytical framework of intimate and peripheral relationships. The exclusion concept provided the basis for our abductive analysis.

### Ethical considerations

Members of the research team recruited the participants in co-operation with local care providers. The participants were assured that they were free to express their thoughts openly and that the interview would have no effect on the services they received. Most of the participants were interviewed once, but one was interviewed twice, having requested a second meeting. The interviews lasted less than two hours, and the researchers always asked the interviewees if they wanted to pause or end the interview before all the themes had been covered. The participants were also given the option to leave questions unanswered, and were assured that they could withdraw from the research at any stage. The researchers were constantly aware of the need to protect the participants’ well-being. Moreover, the significance of methodological issues for both participants and researchers was carefully considered: death is a sensitive or even “taboo topic” in research, which could thus be emotionally laden (Lee 1993). At the end of each interview, the participants were asked if a researcher could make a follow-up call a week later to ask how they were doing. The phone calls allowed the researchers to find out whether the interviews had caused emotional distress and to obtain support for the participant if needed. The two researchers had a reflexive discussion after each interview to ensure that ethical aspects were considered throughout the process, during which they also evaluated the participants’ need for support. The research project was approved by the Ethical Review Board in the Humanities and Social and Behavioural Sciences at the University of Helsinki. At the beginning of each interview, the participants were informed about the purposes of the study and the ethical considerations, and were assured of compliance with General Data Protection Regulations (EU 2016/679).

## Results

The analysis revealed various relationships and changes in them, and the separate and different roles of (intimate) family and peripheral relationships (ten Bruggencate et al. 2018) were strongly visible in the data. The participants generally classified their family relationships as intimate, although sometimes they also included long-term friendships. Other social relationships were considered peripheral. Below, we describe the findings related to these two types of relationship. All seven participants are included in the analysis, but examples from the data are best illustrated in the transcripts of six different interviews.

### Intimate relationships

The interviewees commonly named family members as the most important people in their social networks. The most visible role was that of caregiver, typically a spouse. In a couple of instances, an adult child had taken an active role as a family caregiver when the dying person was living in a single household. Therefore, approaching death affected the social relations of the dying person and their family members the most, as caregiving became an integral part of all family relationships. Caregiving and the responsibilities it entailed included both concrete and practical help, as well as comprehensive care and concern for the dying person. Various arrangements had to be made to ensure that the dying had the assistance they needed at all times, such that different family members became involved during the absence of the primary caregiver. Consequently, care needs brought out elements that had not previously been part of the family relationship. Established roles, responsibilities and tasks changed, particularly between spouses but also between adult children and their parents. The dying person’s care needs also raised expectations related to family relationships and receiving help. These included aspirations to establish closer relationships, particularly with adult children, with a view to having more visits and receiving more assistance:

Maili: When my husband was having that choir practice, Juha visited me because I gave up the car so it was no longer here for me to go and do shopping and other things. Juha took care of things that week when my husband was away at choir practice. Even if [*the other son*] Jari is close by, he doesn't want to [*help, be in contact]* I don’t really understand.

Approaching death also affected interactions among family members: it was a new topic that families needed to discuss. Some families found ways of talking about it. The communicative approach was often co-created during the interactions and reflected certain values or meanings attributed to death within the family. For example, some participants said that there was no need to talk about death, or pointed out that death was discussed openly but matter-of-factly, creating distance from sadness or more emotional responses. However, complicated unspoken feelings associated with death could be challenging and create distance between family members:

Interviewer: How does your wife feel about this latest stage of your illness?

Juhani: Well, it’s one of those things that she never talks about. I just noticed that she takes tranquilisers. Pure accident, we were just in (name of a foreign country) and I had, in my opinion, packed enough medicine. It turned out that there wasn’t enough, so she gave me her own...

We also discovered that relationships do not inevitably change as death approaches. In particular, difficult, broken and distant family relationships are not repaired, nor do they become close, but continue to be overshadowed by previous difficulties within the family. Consequently, changes in family relationships reflected the histories of the families. If the relationships had previously involved conflict, approaching death did not change this. Moreover, communication and interaction among family members did not necessarily increase. The divided nature of family life among different generations accentuated the lack of connection in some families, even when there were no significant challenges in the relationships. If family members lived relatively close, their visits to and social interaction with the dying person could be infrequent. Such distant relationships and the lack of visits or assistance often caused pain:

Maili: Jari lives nearby, but his nature is one of those things, I don’t know. Ever since he was a kid, but now somehow, in some way, he doesn’t want to be in any way, how can I say… Even if it’s my own child, he doesn’t really want to be close to us like that. Even though he’s right there, he’s different in character. But Juha is very... When my spouse wasn’t here and I was here by myself, Juha came to see me every day and brought food and stuff, but Jari not at all.

Tyyne: And then I really refused to fight, I don’t usually do that. Then I wished him happy birthday and happy name day in text messages. Well, thank you, they answer, but that’s all. And the grandchildren, there is no connection there either. I would send them text messages as well, but I start to do it and I don’t have their number. I call directory enquiries, but they can’t give me the number. That is an infinitely painful thing… It still hurts, but I will keep on doing it, when I turned 70 I sent him the invitation, and I will send it again now, but it isn’t up to me whether he comes or not.

Nevertheless, some previously close family relationships remained and brought significant comfort to the dying person. More specifically, a family member with professional expertise, combined with availability, is able to support the dying person by providing them not only with a safety net, but also with much needed advocacy:

Maria: But the fact that I’m so privileged because of my family and my daughter, I don’t need to wait in line at the health centre. […] I am satisfied. My care comes from her [daughter]. Many older people here are in a much worse position. I’m pretty privileged in that sense. Just because I have this family and all of this.

Family members with medical expertise, for example, are able to provide concrete care and assistance as well as to ensure access to treatment and the right kind of medication.

### Peripheral relationships

The interviewees also described other kinds of social contacts they maintained in addition to intimate family relationships. For many, long friendships were among the most important social resources. They brought joy and a sense of meaningfulness to the dying person’s everyday life, even if the number of contacts and friends had decreased:

Anja: And then I have one friend, and our calls can be, and almost often are, an hour to two hours long. We discuss our lives and the way the world is going, and we work in such a way that when one says something, the other can continue the phrase so that you know what the other is talking about, you get along so easily. And it’s going to take so long on the phone, time just flies.

In addition to maintaining long friendships, keeping in contact with former workmates was mentioned as important by many participants. Some former workmates maintained regular contact with them after retirement, and sometimes even thinking about workmates or colleagues was a source of joy and aroused positive feelings.

New relationships may also be formed towards the end of life. Information technology facilitates social interaction and staying in touch with people even when face-to-face meetings are no longer possible:

Anja: Well... It can be very small. When I look at my tablet and there are all these groups, I will add comments in those groups. And when people like things, I make very brief comments there that I’m the one that’s at the heart of the matter, and they’re very often commented on, so I laugh at them all, a bit of funny stuff all. So even a small thing can be welcome.

Previously distant relationships may also become more meaningful, and new supportive relationships, including peer-to-peer friendships, may be initiated. Sometimes, however, the dying person might voluntarily and consciously initiate withdrawal from social relationships:

Interviewer: Is it the case, though, that you have always found a person you could talk to if you wanted to talk, whether it’s your wife or daughters, or a doctor, or researchers?

Henrik: Yes, it is, of course. I'm not exactly abandoned, even though I’ve put a few relationships on hold.

Communication in previously close and meaningful relationships in different communities may be less frequent for other reasons, such as discretion, which may leave the dying person and the caregiver feeling isolated. At times the caregiver regulated the maintenance of social relationships, or help from others, for example, acting as a gatekeeper for social interaction. However, the presence of the caregiver shielded the dying person from feeling alone.

Supportive and help-giving social networks could offer concrete assistance, such as sharing care-taking roles within the family. In addition, they could meet the social needs of both the dying person and the caregiver: the maintenance of social ties supports the mutual relationship between them.

However, the participants mostly referred to a growing distance in non-intimate relationships. They described various social networks to which they used to belong through work, hobbies, friendships and family relations. Some had had particularly active social lives. With the approach of death, however, social relationships underwent changes, and many social contacts weakened:

Maili: Yes, it has changed a lot for me so we had a lot of friends like that, so we were together and we had fun together, but now we’re not. They’re older, too, and maybe it’s mutual. You can see these old friends and friends at church on Sundays. A little chat with them, but they’re also kind of like that, living their own lives, when they’re lonely and things like that, I’m not the closest friend they had. So maybe because those people change and...

In many cases, approaching death reduced the opportunity to socialise via informal relationships, which negatively affected the maintenance of social relationships. Weakening physical capacities and the need for care prevented participation in social activities. The interviewees described feelings of fatigue that limited their ability to tolerate particularly burdensome social interactions, therefore they kept to themselves:

Maria: I don’t try to keep in touch with people, I can’t do it anymore. I was in a very good working community, […] but the people have died with whom... that working community was unbelievably good. We had things to do all the time. It was after retirement, but a few moved to other cities, or then they’re dead. With my illness now, it’s so hard to move that I’m not so much out there. I don’t even want to be anymore...

Moreover, reactions to approaching death negatively affected some social contacts. The dying as well as their caregivers recalled that sometimes friends and other people could not pay them a visit because they felt too uncomfortable and scared.

## Discussion

Approaching death affects the social relations of the dying. In our study, we identified processes of exclusion from existing relations. The dynamic nature of exclusion (Walsh et al. 2021) was strongly present in the data. On the other hand, new and sometimes unexpected relations were initiated, which became meaningful and supportive.

The transition of the relationship between the dying person and the caregiving family member into a care relationship increased the risks of exclusion due to dependence and the perceived burdensomeness of the care. Although the physical bond between the dying person and their family caregiver often became stronger, the emotional distance might grow. Additionally, symbiotic features emerged in relationships involving care needs.

A major aspect was the question of expectations and how social relations met them. Expectations, in turn, are connected with the relative nature of exclusion: they arise from what is considered to be adequate in society (Burholt and Aartsen 2021). Even when intimate relationships existed, they were not necessarily fulfilling. Unmet needs and expectations embedded in family relationships were highlighted with the approach of death. In particular, expectations of receiving care from family members were sometimes unfulfilled, despite attempts to rationalise and explain away their needs and the lack of help by emphasising the hectic lifestyles of adult children, for example. Nevertheless, inherent in such explanations were expectations of connection and support from family members. Our results match findings reported in other studies indicating that older adults in Finland worry about and, to some extent, hope for care from their family members (Outila et al. 2019). However, many families do not discuss end-of-life matters*.* They do not explicitly plan for end-of-life care and dying, even if such questions occupy the thoughts and reflections of older adults (Outila et al. 2019: 118).

According to our data, a lack of communication within families regarding different aspects and feelings connected to death and dying increased emotional distance. Rather than bringing people together, the approaching death could also reactivate difficult family histories and bring to the surface negative experiences or conflicts that had gone unresolved, which in turn increased the distance between family members and prevented interactions among them. The absence of functioning intimate relationships and networks of care and support increased the risk of exclusion when facing death. This finding underlines the importance of analysing exclusion from a life-course perspective (Ejlskov et al. 2019). Long-lasting adversities in family histories were strongly present in our data, and approaching death did not delete the experiences: on the contrary, the problems were experienced as even more painful in the current life situation.

However, social support and reciprocity among family members seemed to decrease the risk of social exclusion. More specifically, satisfying and well-functioning intimate relationships, and the possibility to use their professional expertise to meet the needs of the dying person constituted the most effective forms of support from family members. The shared life situation sometimes also strengthened and deepened relationships, as families found their own ways of dealing with and communicating about dying. Furthermore, the needs of the dying caused changes in family dynamics, and in family practices (Chambers et al. 2009), which were re-negotiated in adapting to the new situation.

In terms of non-family (peripheral) relationships, the participants described how approaching death increased the risk of feeling lonely and isolated. Exclusion from meaningful relationships resulted as previously close contact became less frequent or stopped completely. Such changes were mainly attributable to the dying person’s decreasing physical and mental capabilities, which did not support maintaining contact. Exclusion was especially apparent in social relationships related to activities outside the participants’ homes. It was also apparent if the approach of death scared other people, if former friends and acquaintances distanced themselves out of discretion, or if they were unable to handle their own feelings when meeting and communicating with a dying person. In some cases the caregiver acted as a gatekeeper and reduced social contacts to protect the dying person, sometimes without taking into consideration the person’s own wishes.

On the other hand, facing death in the near future could even decrease the risk of exclusion from social relationships. As mentioned earlier, the presence of the caregiver meant that the dying person was not alone. Moreover, previously loose relationships could become tighter and become meaningful, and new, supportive relationships, including on the peer-to-peer level, were even initiated during the dying process.

Finally, we found that spending more time alone could be experienced as a positive factor in terms of well-being instead of increasing feelings of exclusion from social relationships, which is attributable to voluntary withdrawal, also known as solitude (Nguyen et al. 2017). The participants pointed out that it gave them the possibility to rest and to adapt to the new life situation without social pressure. Voluntary withdrawal from social relationships could also reflect being more selective in one’s choice of social and other activities. This is one of the main premises in the theory of gerotranscendence (Tornstam 2005) in old age, according to which people evaluate the importance of relationships, wishing to distance themselves from those that are burdensome and less meaningful.

## Conclusions

It became apparent during our study that dying is not only a physical process. The ending of life in old age—knowing that life will end in the near future due to an incurable illness—is a social process as well, which includes both increasing and decreasing risks of exclusion from social relationships. Approaching death also affects the social relations of both the dying and the care givers. Therefore, the social dimensions of the process should also be taken into account in the development of policies that affect the organisation of support for a dying older adult. Indeed, the social needs of dying people and their care givers should be considered in the system to include different services connected to death and dying. The focus, therefore, should be not only on physical care, but also on supporting both dying people and caregivers, the relationship between them and the possibilities of maintaining social relationship as death approaches.
