# Correction: Social relations and exclusion among people facing death

**DOI:** 10.1007/s10433-023-00754-1

**Published:** 2023-03-13

**Authors:** Marjaana Seppänen, Mia Niemi, Sofia Sarivaara

**Affiliations:** University of Helsinki, Helsinki, Finland

**Correction to: European Journal of Ageing (2023) 20:1**

**https://doi.org/10.1007/s10433-023-00749-y**

Following publication of the original article [1], the authors would like to change the corresponding author from Mia Niemi to Marjaana Seppänen.

The original article has been corrected.

